# Regional Emerging Diseases Intervention (REDI) Centre

**Published:** 2011-01-10

**Authors:** Rodney Hoff, Ai Li Quake, Za Reed, Rohini Rao, Yeean Leng Kong, Soo Sim Lee, Philippe Cavailler

**Affiliations:** 1 Regional Emerging Diseases Intervention (REDI) Centre, Singapore

## Genesis

The Regional Emerging Diseases Intervention (REDI) Centre is a research and training organization, founded by Singapore and the United States to enhance the Asia-Pacific region’s capability and capacity to deal with emerging and reemerging infectious diseases. REDI Centre is chartered in Singapore as a non-profit, intergovernmental Organization.

## Missions

i/ To promote international exchange of information, knowledge and expertise through the organization of scientific conferences, ii/ to act as a regional resource for training and research, iii / to support programs that will strengthen the response and the control to critical health threats, and iv / to improve regional capacity to prevention, surveillance diagnosis and management of Emerging Infectious Diseases (EIDs).

## Regional conferences recently organized

Forum on Hand Foot and Mouth Disease, Chikungunya Symposium and Fourth ASEAN Congress on Tropical Medicine and Parasitology.

## Laboratory capacity

Together with Singapore and international partners (WHO, US-NIH …), REDI Centre organized several on-site lab training in Singapore public health laboratories as well as regional trainings on laboratory techniques and principles for EIDs diagnosis (biosafety train-the-trainer courses, Influenza PCR and virus isolation and characterization workshops, Asia-Pacific Dengue workshops). REDI collaborated with partners to form a regional network with the aim to improve laboratory EIDs diagnosis and surveillance (biosafety, quality insurance, development of guidelines for EID diagnosis).

## Clinical capacity

In the area of hospital infection control, REDI Centre actively contributed to the development of state-of-the-art training modules and currently assists the MOHs of Vietnam (implementation of the National Infection Prevention and Control Master Plan), Indonesia (revision of the National Guideline for AI management and infection control) and Cambodia (surveillance of nosocomial infections in Kampong Cham Hospital).Together with Pasteur Network, WHO and other partners, organization of a regional course on Infection Control in Vientiane (with Pasteur Network) and development of clinical guidelines for the clinical management of Hand, Foot and Mouth Disease

## Epidemiology capacity

REDI centre supports Singaporean and Indonesian FETPs and co-organized (with Pasteur Network and National University of Singapore) several short regional courses:“Data-management and Basic Statistics”, “Outbreak Investigation”. In Indonesia REDI Centre collaborates in the Trilateral Pilot Project on Surveillance and Control of Avian Influenza in Tangerang (with Indonesian Ministries of Health and Agriculture) and in the realization of a study aiming to assess the H5N1 seroprevalence in poultry workers and in poultry in live market facilities (with US-CDC and NIHRD).

